# Safety and patient experience with at‐home infusion of ocrelizumab for multiple sclerosis

**DOI:** 10.1002/acn3.51745

**Published:** 2023-02-22

**Authors:** Britney Barrera, Haley Simpson, Eric Engebretson, Stefan Sillau, Brooke Valdez, José Parra‐González, Ryan C. Winger, Lou Anne Epperson, Ashley Banks, Kathryn Pierce, Melanie Spotts, Katie O'Gean, Enrique Alvarez, Robert Gross, Amanda L. Piquet, Teri Schreiner, John R. Corboy, Jinglan Pei, Timothy L. Vollmer, Kavita V. Nair

**Affiliations:** ^1^ Department of Neurology, Rocky Mountain MS Center University of Colorado School of Medicine Aurora Colorado USA; ^2^ Genentech, Inc South San Francisco California USA; ^3^ Amerita Specialty Infusion Services Denver Colorado USA; ^4^ University of Colorado Hospital Aurora Colorado USA; ^5^ Skaggs School of Pharmacy and Pharmaceutical Sciences University of Colorado Anschutz Medical Campus Aurora Colorado USA

## Abstract

**Objective:**

This study aimed to evaluate safety (infusion‐related reactions [IRRs]) and patient satisfaction (patient‐reported outcomes [PROs]) for at‐home ocrelizumab administration for patients with multiple sclerosis (MS).

**Methods:**

This open‐label study included adult patients with an MS diagnosis who had completed a ≥ 600‐mg ocrelizumab dose, had a patient‐determined disease steps score of 0 to 6 and had completed PROs. Eligible patients received a 600‐mg ocrelizumab home‐based infusion over 2 h, followed by 24‐h and 2‐week post‐infusion follow‐up calls. IRRs and adverse events (AEs) were documented during infusions and follow‐up calls. PROs were completed before and 2 weeks post infusion.

**Results:**

Overall, 99 of 100 expected patients were included (mean [SD] age, 42.3 [7.7] years; 72.7% female; 91.9% White). The mean (SD) infusion time was 2.5 (0.6) hours, and 75.8% of patients completed their ocrelizumab infusion between 2 to 2.5 h. The IRR incidence rate was 25.3% (95% CI: 16.7%, 33.8%)—similar to other shorter ocrelizumab infusion studies—and all AEs were mild/moderate. In total, 66.7% of patients experienced AEs, including itch, fatigue, and grogginess. Patients reported significantly increased satisfaction with the at‐home infusion process and confidence in the care provided. Patients also reported a significant preference for at‐home infusion compared with prior infusion center experiences.

**Interpretation:**

IRRs and AEs occurred at acceptable rates during in‐home infusions of ocrelizumab over a shorter infusion time. Patients reported increased confidence and comfort with the home infusion process. Findings from this study provide evidence of the safety and feasibility of home‐based ocrelizumab infusion over a shorter infusion period.

## Introduction

Treatment for multiple sclerosis (MS) often requires routine and long‐term care for optimal disease management, including the administration of disease‐modifying therapies (DMTs) by intravenous (IV) infusion.^1^, ^2^ Due to the high costs of DMT infusions^3^, ^4^ and patient treatment satisfaction,^5^, ^6^, ^7^, ^8^ payers have increasingly implemented different site of care (SOC) strategies, including mandating treatment at outpatient and non‐hospital‐affiliated infusion centers, and reducing infusion times, to manage infusion costs and treatment accessibility. In 2018, 61% of payers reported the use of SOC management policies, in which patients were directed to the payers' preferred outpatient infusion sites to receive treatment; this was an increase from 39% in 2017.^9^ This SOC strategy has had a large impact on patients' access to IV DMTs, notably increasing the use of independent infusion sites at the expense of academic centers. Evaluating the feasibility and benefit of this trend was accelerated during the COVID‐19 pandemic to ensure that patients with MS could continue treatment while adhering to health and safety precautions,^10^, ^11^, ^12^ including shorter, at‐home infusions.^13^

Ocrelizumab is a recombinant, humanized monoclonal antibody that selectively targets CD20‐expressing B cells and is approved for the treatment of both relapsing (RMS) and primary progressive (PPMS) forms of MS.^14^ The current approved US label indicates ocrelizumab administration of a 600‐mg dose via IV infusion; the first dose is administered as two 300‐mg infusions, separated by 14 days, over a period of 2.5 h or longer.^1^ Subsequent doses of ocrelizumab are given as single 600‐mg infusions either over 3.5 h or over a shorter 2‐h duration if no serious infusion‐related reactions (IRRs) occurred with any previous infusion.

A number of recent clinical studies have evaluated the safety of shorter infusion times for patients with MS who received ocrelizumab treatment.^15^, ^16^, ^17^ The safety profile established in the pivotal phase III OPERA I (NCT01247324), OPERA II (NCT01412333), and ORATORIO (NCT01194570) studies found that ocrelizumab IV administration, which followed the conventional 3.5‐h infusion time, is generally well tolerated, with IRRs reported as the most common adverse event (AE).^18^, ^19^, ^20^, ^21^, ^22^ More recently, when compared with the conventional 3.5‐h ocrelizumab infusion time at infusion centers, the shorter 2‐h infusion resulted in similar rates of IRRs in the ENSEMBLE PLUS extension (NCT03085810) and CHORDS (NCT0237856) substudy, respectively.^15^, ^16^ All IRRs reported with an ocrelizumab infusion were mild or moderate, and no serious IRRs were reported across studies. Additionally, the ShoRter Ocrelizumab infusion stuDy (SaROD) (NCT03606460) further substantiated the acceptable safety findings achieved with a reduction in infusion time at an infusion center compared with the conventional infusion protocol in patients with MS.^17^

The safety and benefit of the reduced infusion time for ocrelizumab has been established; however, limited data are available for the safety of home‐based infusions, especially in MS. The aim of this study was to develop and investigate a feasible and safe home infusion model, as well as to gain insights into the patient experience, for those who receive at‐home infusions of ocrelizumab and are monitored by telehealth visits for translation into routine clinical practice.

## Methods

### Trial design

This was an open‐label, single‐arm, nonrandomized study to evaluate the safety and patient experience of ocrelizumab infusion over a 2‐h period via home‐based infusion (NCT04650321). The similarly designed Phase IIIb, open‐label SaROD study of accelerated ocrelizumab infusions was used as a historical control.^17^

**Figure 1:**
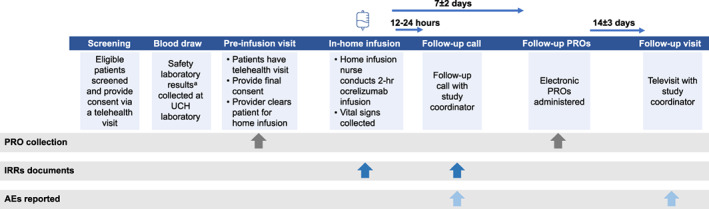
Study design. AE, adverse event; CBC, complete blood count; CMP, comprehensive metabolic panel; Ig, immunoglobulin; IRR, infusion‐related reaction; PRO, patient‐reported outcome; UCH, University of Colorado Health. ^a^ Therapy monitoring laboratory results collected prior to home infusion included CBC, CMP, Ig panel (IgG, IgA, IgM) and blood pregnancy test.

Eligible patients were contacted by study research coordinators and screened for potential study recruitment, then provided preliminary electronic consent through a telehealth visit (Fig. 1). Patients were required to undergo a blood draw at any of 23 UCHealth laboratory locations convenient for them in order to assess eligibility for home infusion. A telehealth visit with an MS neurologist to discuss laboratory results, questions, concerns and benefits of a home infusion was then conducted, at which point final consent was obtained, and patients were sent electronic patient‐reported outcome (PRO) forms to assess their experiences of prior non‐home‐based ocrelizumab infusion. The at‐home infusions were conducted by nurses employed by Amerita Specialty Infusion Services. A pre‐medication protocol was developed to minimize the frequency and severity of IRRs. This included the administration of 125 mg methylprednisolone IV along with 50 mg diphenhydramine either IV or orally and 650 mg of acetaminophen orally with the option to add an oral antihistamine, 30 to 60 min prior to start of infusion.

Patient vitals, IRRs and any AEs were documented throughout the infusion process. Following the home infusion, research coordinators called the participants within 24 h to follow‐up on any additional AEs that they may have experienced. Electronic PRO forms similar to those completed prior to home infusion were sent a week later to assess the patient's experience with the home infusion and to reassess their general health. A final telehealth study visit was conducted with a research coordinator 2 weeks after the home infusion to collect any additional ocrelizumab‐related AEs and to close the study.

This study was conducted in accordance with the current US Food and Drug Administration Good Clinical Practices and local ethical and legal requirements. All study participants provided informed consent via electronic signature prior to participation in the study.

### Patient population

In this study, participant selection was designed to reflect real‐world patients with MS who receive ocrelizumab. Enrollment occurred on a rolling basis over a 12‐month period from active patients of the Rocky Mountain MS Center (RMMSC) at the University of Colorado Anschutz Medical Campus until the target population of 100 patients was met for the intention‐to‐treat population (ITT).

Eligible patients included adults aged 18 to 55 years who: (1) had a diagnosis of RMS or PPMS as defined by the 2017 McDonald criteria;^23^ (2) completed a minimum of their first 600‐mg ocrelizumab dose; (3) physically resided in an area with access to emergency services (eg, 911); (4) provided consent to the use of an effective form of contraception throughout the study; (5) had a Patient Determined Disease Steps (PDDS) score of 0 to 6;^24^ (6) treating Continuous usage of ocrelizumab was deemed appropriate medically appropriate by their treating RMMSC neurologist; and (7) were able to complete PROs in English at the time of the final study consent.

Patients were excluded from participation in this study if they experienced a serious IRR (>Grade 3) during a prior ocrelizumab infusion, were pregnant on the day of infusion or intended to become pregnant before study completion, were breastfeeding at time of final study consent or before the end of the study, and had participated in the SaROD trial at the RMMSC site or if their most recent ocrelizumab infusion prior to consent was a home infusion.

### Outcome measures

Data collected at screening and pre‐infusion visits consisted of patient demographics (age, sex, race, and ethnicity) and clinical disease characteristics (type of MS, disease duration, DMT type, DMT duration, PDDS score, and number of prior ocrelizumab infusions). Infusion procedure variables documented during the infusion included pre‐infusion medications, time to complete the infusion, ocrelizumab dose (mg and mL).

The primary outcome was the proportion of patients with IRRs in this study, and in the SaROD historical comparator cohort 1. The SaROD cohort 1 study enrolled patients who had completed ≥1 dose of 600‐mg ocrelizumab according to the US label prior to study screening, then received an additional dose of ocrelizumab 600 mg over the shorter infusion time, as planned with our study design. Severity of IRRs was determined using the Common Terminology Criteria for Adverse Events developed by the National Cancer Institutes and utilized in the OPERA I and II studies.^18^, ^19^ IRRs that occurred during patient infusion were classified as Grade 0 (no IRR), Grade 1 (mild reaction [transient flushing or rash; drug fever <38°C], infusion interruption not indicated or intervention not indicated), Grade 2 (reaction that requires therapy or infusion interruption, but responds promptly to symptomatic treatment [eg, antihistamines, nonsteroidal anti‐inflammatory drugs or narcotics] or prophylactic medications indicated for 24 h), Grade 3 (prolonged [ie, not rapidly responsive to symptomatic medication and/or brief interruption of infusion], recurrence of symptoms following initial improvement or hospitalization indicated for other clinical manifestations [eg, renal impairment, pulmonary infiltrates]), Grade 4 (life‐threatening consequences or urgent intervention indicated), and Grade 5 (death).

Secondary outcomes included PRO measures of patient experiences with home infusion and number of patient‐reported adverse events (AEs) (Supporting information Appendix S1). The same measures were used to assess patient recall of their experience at their last ocrelizumab infusion administered at an infusion center to evaluate pre‐ and post‐infusion processes. PROs included adapted versions of the Hospital Consumer Assessment of Health systems and Providers Surveys and the Consumer Assessment of Healthcare Providers and Systems Home Healthcare Survey^25^, ^26^; questionnaire topics included overall rating of the home infusion experience, confidence in nurses, feelings regarding respect and safety and comfort with surroundings (all scale 0–5; higher score corresponds to better experience); clarity of information provided by and treatment received from infusion nurses (both scale 0–4, higher score is better); and confidence in receiving an infusion at home or at infusion center (both scale 0–10; higher score denotes positive experience). Additionally, AEs were assessed within the first 24 h post infusion and at the end of 2 weeks through telehealth visits conducted by a research coordinator. Any serious AEs reported were managed by the patient‐treating neurologist at the RMMSC.

### Statistical analysis

Descriptive statistics were used to summarize patient demographics, clinical disease characteristics, infusion procedure variables and AEs for the ITT population. Controls were limited to available historic data from the SaROD study.

The frequencies and proportions of IRRs for the primary outcome were summarized descriptively. The 95% confidence intervals were calculated for IRR proportions; exact methods were used as necessary. Descriptive statistics and 95% confidence intervals for the mean were calculated for the PROs. PRO data were not available for the historical controls. When applicable, pre‐post infusion comparisons of PROs were performed. For continuous or scale outcomes, paired *t* methods were used to calculate *p* values and 95% confidence intervals for pre‐post infusion comparisons.

## Results

### Patient demographics and disease characteristics

**Figure 2:**
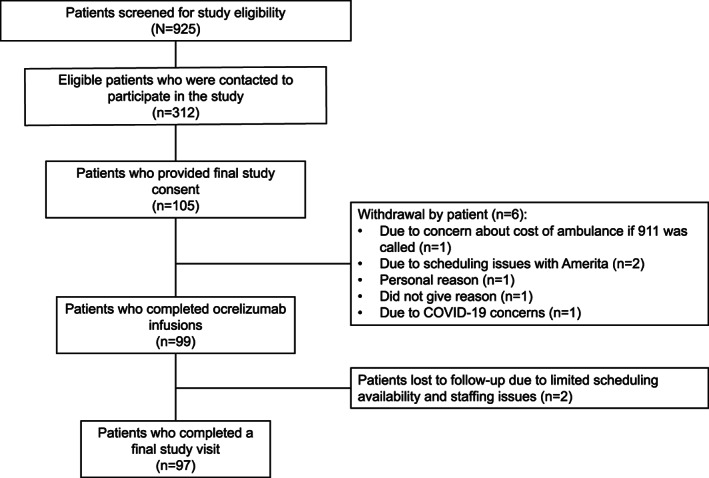
Patient identification and attrition.

Between August 2020 and February 2021 medical charts were reviewed for 925 eligible patients with MS, and 312 patients were contacted by the study team (Fig. 2). A total of 105 patients provided final study consent, 99 of whom completed the study infusions and were included in the ITT population; 97 completed a final study visit.

A total of 99 patients with MS were included in the ITT population in this home infusion cohort study. The mean (SD, range) age was 42.3 (7.7, 25.7–55.5) years, and most patients were female (72.7%) and White (91.9%) (Table 1). This study included patients with RMS (95.8%) or PPMS (4.2%) (data were missing for 3 patients). When referencing the patient population in the SaROD study, comparable demographics were observed.^17^

**Table 1:** Patient demographics and disease characteristics by study cohort.

Characteristic, *n* (%)^a^	Home infusion cohort (*n* = 99)	SaROD cohort 1^17^ (*n* = 95)
Age, mean (SD), years	42.3 (7.7)	41.7 (8.8)
Sex		
Female	72 (72.7)	59 (62.1)
Male	27 (27.3)	36 (37.9)
Race		
White	91 (91.9)	86 (90.5)
Black/African American	4 (4.0)	8 (8.4)
Other	4 (4.0)	–
Multiple	–	1 (1.1)
Ethnicity		
Non‐Hispanic/LatinX	96 (97.0)	88 (92.6)
Hispanic/LatinX	3 (3.0)	4 (4.2)
Unknown	–	3 (3.2)
Weight, mean (SD), kg	80.1 (24.2) [97]^b^	82.2 (19.8) [91]^b^
MS type		
RMS	92 (95.8) [96]^b^	86 (90.5)
PPMS	4 (4.2)	9 (9.5)
Disease duration, mean (SD), months	70.1 (346.9)	–
DMT duration, mean (SD), months	64.1 (347.9)	–
EDSS, median (IQR)		2.00 [1.50–3.00]
<4	–	76 (80.0)
≥4	–	17 (17.9)
Missing	–	2 (2.1)
PDDS, median (IQR)	1 [0–2]	–
<3	79 (79.8)	–
≥3	20 (20.2)	–
No. of prior infusions, mean (SD)	5.6 (1.9)	–

DMT, disease‐modifying therapy; EDSS, Expanded Disability Status Scale; IQR, interquartile range; MS, multiple sclerosis; PDDS, patient‐determined disease steps; PPMS, primary progressive MS; RRMS, relapsing–remitting MS.

^a^ Data presented at *n* (%), unless otherwise stated.

^b^ [*n*] denotes number of patients with available information.

The mean (SD) disease duration and number of prior infusions was 70.1 (346.9) months and 5.6 (1.9) infusions, respectively (Table 1). The median (interquartile range [IQR]) PDDS was 1 [0–2], and 20.2% had a PDDS score of ≥3. The SaROD study measured disease activity with the Expanded Disability Status Scale, and the median (IQR) Expanded Disability Status Scale score was 2.00 [1.50–3.00] in SaROD cohort 1.^17^

### Summary of at‐home ocrelizumab infusion procedures

The majority of patients received acetaminophen (94.9%), methylprednisolone (89.9%) and diphenhydramine (88.9%) prior to their ocrelizumab infusion (Table 2). The overall mean (SD) infusion time was 2.5 (0.6) hours for this cohort, with 75.8% of patients experiencing an infusion time of >2 to 2.5 h. Patients in SaROD cohort 1 had similar infusion encounters, with the majority of patients receiving diphenhydramine (100.0%) and methylprednisolone (90.5%) before infusion.^17^ The mean (SD) infusion time was 2.4 (0.3) hours, and 78.9% of patients also had an infusion time of >2 to 2.5 h.

**Table 2:** Infusion procedure by study cohort.

Variable	Home infusion cohort (*n* = 99)	SaROD cohort 1^17^ (*n* = 95)
Pre‐infusion medication, *n* (%)		
Diphenhydramine (50 mg IV or oral)	84 (88.8)^a^	95 (100.0)
Acetaminophen (650 mg oral)	94 (94.9)	–
Methylprednisolone (125 mg IV)	89 (89.9)	86 (90.5)
Oral antihistamines		
Cetirizine (10 mg)	1 (1.0)	
Loratadine (10 mg)	2 (2.0)	
Infusion time, mean (SD), hours	2.5 (0.6)	2.4 (0.3)
≤1.5	1 (1.0)	–
1.5–2	1 (1.0)	–
>2–2.5	75 (75.8)	75 (78.9)
>2.5–3	6 (6.1)	17 (17.9)
>3^b^	16 (16.2)	3 (3.2)
Ocrelizumab dose, mean (SD), mg	599.9 (0.8)	600.0 (0.0)
Ocrelizumab dose, mean (SD), mL	513.9 (16.9)	564.7 (39.8)

^a^ One patient switched to 25 mg of diphenhydramine due to a prior reaction with the 50‐mg dose.

^b^ Longer infusion times may have been a result of mechanical and other interruptions.

### IRRs with at‐home ocrelizumab infusion administration

**Figure 3:**
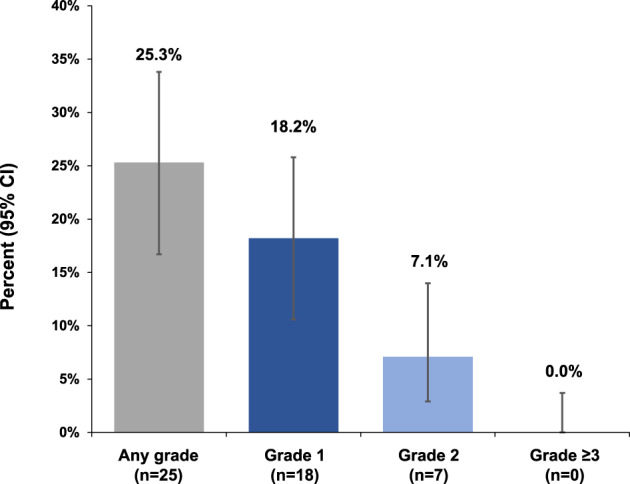
Frequency of infusion‐related reactions experienced with home infusion by grade.

Overall, only 25.3% (95% CI: 16.7%, 33.8%) of patients experienced an IRR of any grade during the at‐home ocrelizumab infusion. These IRRs were 18.2% Grade 1 (95% CI: 10.6%, 25.8%) and 7.1% Grade 2 (95% CI: 2.9%, 14.0%); no IRRs were classified as ≥Grade 3 (95% CI: 0.0%, 3.7%) (Fig. 3). A numerically lower proportion of patients experienced an IRR in this study population compared with the SaROD cohort 1 population for any‐grade IRRs, as well as those determined to be Grade 1 and 2; no ≥Grade 3 IRRs were reported (Table 3).

**Table 3:** IRR frequency by study cohort.

IRR worst grade per patient, *n* (%) [95% CI]	Home infusion cohort (*n* = 99) (%)	SaROD cohort 1^17^ (*n* = 95) (%)
Total IRRs, *n*	25	65
No IRR	74 (74.8) [66.2, 83.3]	49 (51.6) [41.5, 61.6]
Any grade IRR	25 (25.3) [16.7, 33.8]	46 (48.4) [38.4, 58.5]
Grade 1	18 (18.2) [10.6, 25.8]	32 (33.7) [24.2, 43.2]
Grade 2	7 (7.07) [2.89, 14.0]	14 (14.7) [7.6, 21.9]
Grade ≥3	0 (0.0) [0, 3.7]	0 (0.0) [0, 3.8]

IRR, infusion‐related reaction.

### Patient‐reported AEs that occurred with at‐home infusion

**Figure 4:**
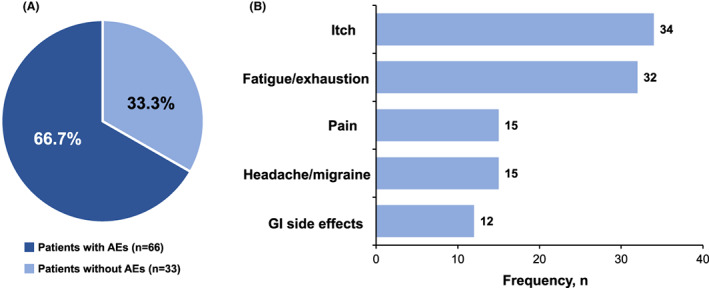
Post‐infusion (A) patient‐reported AEs and (B) most common patient‐reported AEs. AE, adverse event; GI gastrointestinal.

Among these patients, a total of 174 AEs were reported, and 66.7% of patients experienced an AE with at‐home ocrelizumab infusion (Fig. 4A). The most commonly reported AE was itch, followed by fatigue and grogginess, pain, headache/migraine, and gastrointestinal AEs (Fig. 4B). No serious AEs were reported post‐infusion.

### Patient experience with home‐based ocrelizumab treatment

**Figure 5:**
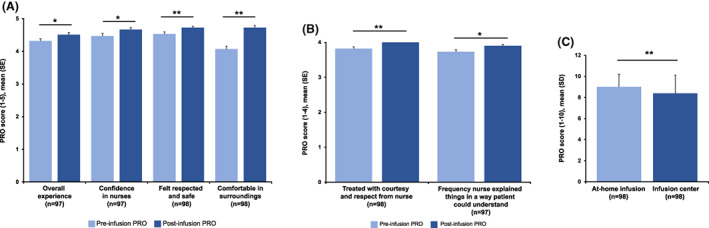
The (A) overall patient experience with ocrelizumab home infusion, (B) patient experience with nurses during home infusion and (C) patient preference for infusion location.^a^ PRO, patient‐reported outcome. ^a^ Higher PRO score is indicative of a more positive experience for the patient. *p < 0.05, **p < 0.01.

Patients reported favorable scores across PRO measures of their at‐home infusion experience, compared to their prior infusion experience at an infusion center. Significant improvements from were reported following a home for overall experience with the at‐home infusion process (*p* = 0.0409), including patient confidence in their nurse (*p* = 0.0109), feeling respected and safe during the infusion (*p* = 0.0041) and being comfortable in their surroundings (*p* < 0.0001) (Fig. 5A). Post home infusion, patients also reported high satisfaction with being treated with courtesy and respect from their home infusion nurse (*p* = 0.0023) and felt that their nurse frequently explained things in a way they would understand (*p* = 0.0449) (Fig. 5B). Patients also reported a better experience with their at‐home infusion of ocrelizumab compared with their prior treatment at an infusion center (*p* = 0.0046) (Fig. 5C).

## Discussion

This open‐label, single‐arm, nonrandomized study is among the first to assess the safety of the at‐home infusion of ocrelizumab. Leveraging the shorter infusion regimen, at‐home administration did not increase the incidence of IRRs compared with the SaROD trial of the shorter infusion protocol for administering ocrelizumab, and no cases of severe or life‐threatening IRRs were experienced, which highlights the safety of at‐home infusions. Overall, patients reported increased confidence and comfort during home infusions through PROs administered pre‐ and post‐infusion compared with their last infusion received at an infusion center.

Our study is among the first to evaluate home infusion for the administration of ocrelizumab, and findings from this cohort provide evidence of the safety, feasibility and patient satisfaction that can be achieved with at‐home infusions. Our study supports the use of home infusions as another safe and efficient alternative option for administering ocrelizumab to stable MS patients. Limited data on the administration of at‐home infusions are available, which highlights the importance of these study findings as providers of MS patients find newer ways to administer ocrelizumab. It is important to note that available studies in other DMTs and antibiotic treatment administration have shown comparable safety and efficacy with at‐home treatments compared with standard clinical settings. In a pilot crossover clinical study, patients with MS were randomized to receive usual care or home infusions of natalizumab, and were then switched to receive the alternate treatment.^27^ No AEs were recorded in the crossover pilot study of natalizumab treatment, and no differences were found between adherence or infection rates between settings. Significantly greater satisfaction with convenience was found with home infusions, and costs were reduced compared with infusions received in clinical settings. Additionally, a systematic review of studies that compared home‐ and hospital‐based infusions of IV antibiotics for quality, safety and patient satisfaction demonstrated that patients who received home‐based care were not more likely to report AEs and had comparable clinical outcomes compared with those who received hospital‐based infusions.^13^ Relatively small sample sizes and the differences in the nature of these studies may limit the scope of comparison with our study; however, they do add evidence to support the general feasibility of home‐based treatments without compromising patient safety.

The mean infusion time achieved in this study was 2.5 h, which is consistent with the historical control data of 2.4 h reported in the SaROD study.^17^ The low IRR incidence rate of 25.3% seen in our study is comparable with or lower than that from previously published studies with shorter infusion times. In SaROD cohort 1, the shorter infusion administration time provides the opportunity to substantially reduce treatment burden associated with ocrelizumab that patients and providers experience; it also offers the chance to maintain social distancing that some patients with MS may need due to the ongoing COVID‐19 pandemic and future pandemics. In total, 48.4% of patients experienced an IRR. In the ENSEMBLE PLUS substudy of the original ENSEMBLE clinical trial, 28.8% of patients in the shorter infusion group experienced IRRs compared with 26.5% of patients in the conventional infusion group,^16^ while experiencing a median reduction in infusion time of 95 min.

The findings from this study are also unique because they provide valuable insights into the patient experience with at‐home infusion treatments through PRO questionnaires. The increased use of PRO scales in clinical studies enhances our understanding of patients' feelings about the treatment experience, their interactions with healthcare providers and circumstances that may influence their adherence to treatment. A survey study of patients with MS who received treatment at an infusion center reported that 83% of patients who received natalizumab or ocrelizumab had to take time off work or study for treatment infusions, and 60% responded that they would consider home‐based infusions.^7^ While these studies are limited in scope due to the different outcomes measured, they provide insight into general patient preferences regarding MS treatment and demonstrate factors that may influence treatment decisions, particularly the dissatisfaction surrounding infusible DMTs and inconvenience with administration. In our study, patients reported improved satisfaction, increased confidence and comfort with at‐home ocrelizumab infusions, which could be attributed to both the reduced treatment time and convenience of home‐based therapy. Significant improvements were also reported for interactions with the nursing staff who administered the infusion and offered explanations throughout the process, which was achievable at home because of the one‐on‐one care provided. PRO findings from our study offer valuable insight into how home‐based infusions can factor in patient preferences and improve treatment satisfaction.

The results presented here should be interpreted within the context of the study limitations. Patient recruitment methods from a single center may limit the generalizability of these results for the overall MS population. The outcomes from this study were also only evaluated for a single home‐based infusion experience; therefore, further studies over longer periods that include multiple infusions should be conducted to extend these results. Historical control data were also used for comparison in the present study, which may introduce bias, although the SaROD study had similar characteristics in terms of sex, race, age and MS disease type. Studies that include direct comparison arms are needed; nevertheless, the findings of this study are consistent with and comparable to similar prior studies. Additionally, some of the PRO questionnaires included in this report are not validated; however, they do include very specific questions tailored to the home‐based infusion process that can provide valuable insights that established PROs may not address. While there are study limitations to consider, the findings presented here add useful evidence for the use of home‐based infusion therapy. Future studies that are designed to specifically address safety and efficacy concerns regarding at‐home infusions can further substantiate our findings.

Overall, the findings presented in this study demonstrate the safety and feasibility of at‐home infusions for patients with MS. We report IRR and overall AE incidence rates comparable to those reported in other clinical studies involving home‐based infusions. This study also provides positive PRO data regarding the patient experience with at‐home ocrelizumab infusions and reveals factors that contribute to patient treatment satisfaction with infusion therapy. The reduction in time spent undergoing treatment through at‐home infusions can increase convenience and patient satisfaction, as well as positively impact treatment adherence without compromising the clinical benefit.

## Author Contributions

*Acquisition of data*: all authors. *Analysis and/or interpretation of data*: Vollmer, Nair, Sillau, Barrera, Engebretson, Winger, Pei. *Drafting the manuscript*: Vollmer, Nair, Sillau, Barrera, Engebretson, Winger, Pei. *Revising the manuscript critically for important intellectual content*: all authors. *Approval of the version of the manuscript to be published* (*the names of all authors must be listed*): all authors.

## Conflict of Interest Statement

**B. Barrera** has nothing to disclose. **H. Simpson** has nothing to disclose. **E. Engebretson** has nothing to disclose. **S. Sillau** has nothing to disclose. **B. Valdez** has nothing to disclose. **J. Parra‐Gonzalez** has nothing to disclose. **R. C. Winger** is an employee of Genentech, Inc, and a shareholder of F. Hoffmann‐La Roche Ltd. **L. A. Epperson** has nothing to disclose. **A. Banks** has nothing to disclose. **K. Pierce** has nothing to disclose. **M. Spotts** has nothing to disclose. **K. O'Gean** has nothing to disclose. **E. Alvarez** has received compensation for activities such as advisory boards, lectures and consultancy from Actelion/Janssen, Alexion, Bayer, Biogen, Celgene/BMS, EMD Serono/Merck, Genentech/Roche, Novartis, Sanofi and TG Therapeutics; and research support from Biogen, Genentech/Roche, Novartis, TG Therapeutics, Patient‐Centered Outcomes Research Initiative, National Multiple Sclerosis Society, National Institutes of Health and Rocky Mountain MS Center. **R. Gross** has nothing to disclose. **A. Piquet** reports research support and consulting fees from Genentech. Outside of this work, Dr. Piquet reports grants from the University of Colorado and Rocky Mountain MS Center, consulting fees from Alexion, honorarium from Medlink and publication royalties from Springer as a co‐editor of a textbook. **T. Schreiner** has received research funding from the National MS Center, Roche, Rocky Mountain MS Center and Biogen; and consultant fees from Roche and MS Focus. **J. R. Corboy** has received research Support from the National Institutes of Health, Patient‐Centered Outcomes Research Institute, National Multiple Sclerosis Society and Novartis; has served on an advisory committee for Bristol Meyers Squibb and on the editorial board for *Annals of Neurology*; and is a Medical Director at the Rocky Mountain Multiple Sclerosis Center. **J. Pei** is an employee of Genentech, Inc, and a shareholder of F. Hoffmann‐La Roche Ltd. **T. L. Vollmer** has received compensation for lectures and consultancy from Biogen IDEC, Genentech/Roche, Celgene, EMD Serono, Bristol Meyers Squib and Novartis; and has received research support from the Rocky Mountain Multiple Sclerosis Center, Biogen, Actelion, Roche/Genentech, F. Hoffman‐La Roche, Ltd and TG Therapeutics, Inc. **K. V. Nair** National MS Center has served as a consultant to Bristol Myers Squibb, Novartis, TG Therapeutics and PhRMA Foundation. She serves on the speakers' bureau of Sanofi‐Genzyme and Alexion, and she has received research grants from Genentech, PhRMA Foundation, Bristol Myers Squibb and Novartis.

## Funding Information

NIH funding was provided by CTSA grants UL1 TR002535, KL2 TR002534, and TLI TR002533. This study was sponsored by F. Hoffmann‐La Roche Ltd and Genentech, Inc. Support for third‐party writing assistance, furnished by Charli Dominguez, PhD, CMPP, of Health Interactions, Inc, was provided by F. Hoffmann‐La Roche Ltd.

## Supporting information


**Data S1.** Supporting information

## Data Availability

The datasets generated and/or analyzed during the current study are not publicly available as this study involved the merging of data from several different sources (patient‐reported outcomes, laboratory outcomes, telehealth visit data) and different electronic systems (home infusion center, academic medical center) but are available from the corresponding author on reasonable request.
