# Supplementary material for: Safety and patient experience with at‐home infusion of ocrelizumab for multiple sclerosis
Source: Ann Clin Transl Neurol. 2023 Feb 22;10(4):579–88. doi: 10.1002/acn3.51745 (PMC10109340; doi:10.1002/acn3.51745)
Supplement: Supplementary file 1 — Data S1. Supporting information [file ACN3-10-579-s001.docx]

**SUPPLEMENTAL MATERIALS**

**Appendix A: Patient-Reported Outcomes (PROs)**

**Pre-Infusion PROs**

**1. During your last infusion, how often did your nurse treat you with courtesy and respect?**

1 Never

2 Sometimes

3 Usually

4 Always

**2. During your last infusion, how often did your nurse listen carefully to you?**

1 Never

2 Sometimes

3 Usually

4 Always

**3. During your last infusion, how often did your nurse explain things in a way you could understand?**

1 Never

2 Sometimes

3 Usually

4 Always

**4. During your last infusion, did you get information about what symptoms or health problems to look out for after the infusion?**

1 Yes

2 No

**5. Using any number from 0 to 10, where 0 is the worst experience possible and 10 is the best experience possible, what number would you use to rate this infusion experience?**

 0 Worst experience possible

 1

 2

 3

 4

 5

 6

 7

 8

 9

 10 Best experience possible

**Additional Questions:**

Overall, receiving my Ocrevus infusion was a good experience.

o strongly disagree

o disagree

o neither agree or disagree

o agree

o strongly agree

I felt confident in infusion nurses administering my infusion.

o strongly disagree

o disagree

o neither agree or disagree

o agree

o strongly agree

I felt respected and safe throughout the infusion.

o strongly disagree

o disagree

o neither agree or disagree

o agree

o strongly agree

I felt comfortable in my surroundings during the infusion.

o strongly disagree

o disagree

o neither agree or disagree

o agree

o strongly agree

I felt worried about possible safety or adverse events during or after the infusion

o strongly disagree

o disagree

o neither agree or disagree

o agree

o strongly agree

If a safety or adverse event were to occur, I felt the medical professionals would be well- equipped to respond appropriately

o strongly disagree

o disagree

o neither agree or disagree

o agree

o strongly agree

The location of the infusion center is convenient to home/work/daily schedule

o strongly disagree

o disagree

o neither agree or disagree

o agree

o strongly agree

**PROMIS 10 – Global Health**

**Please respond to each question or statement by marking one box per row.**


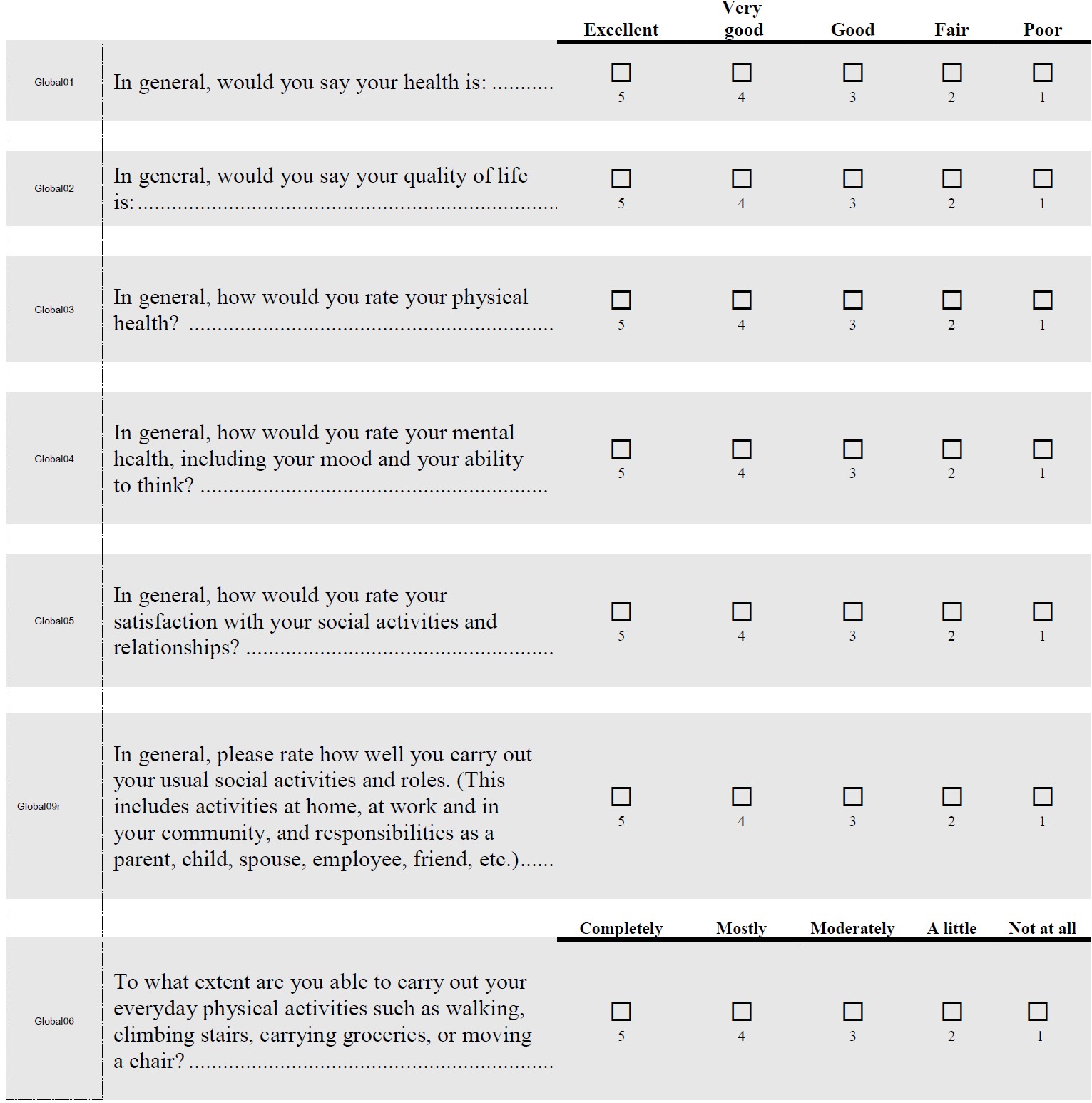


**In the past 7 days…**


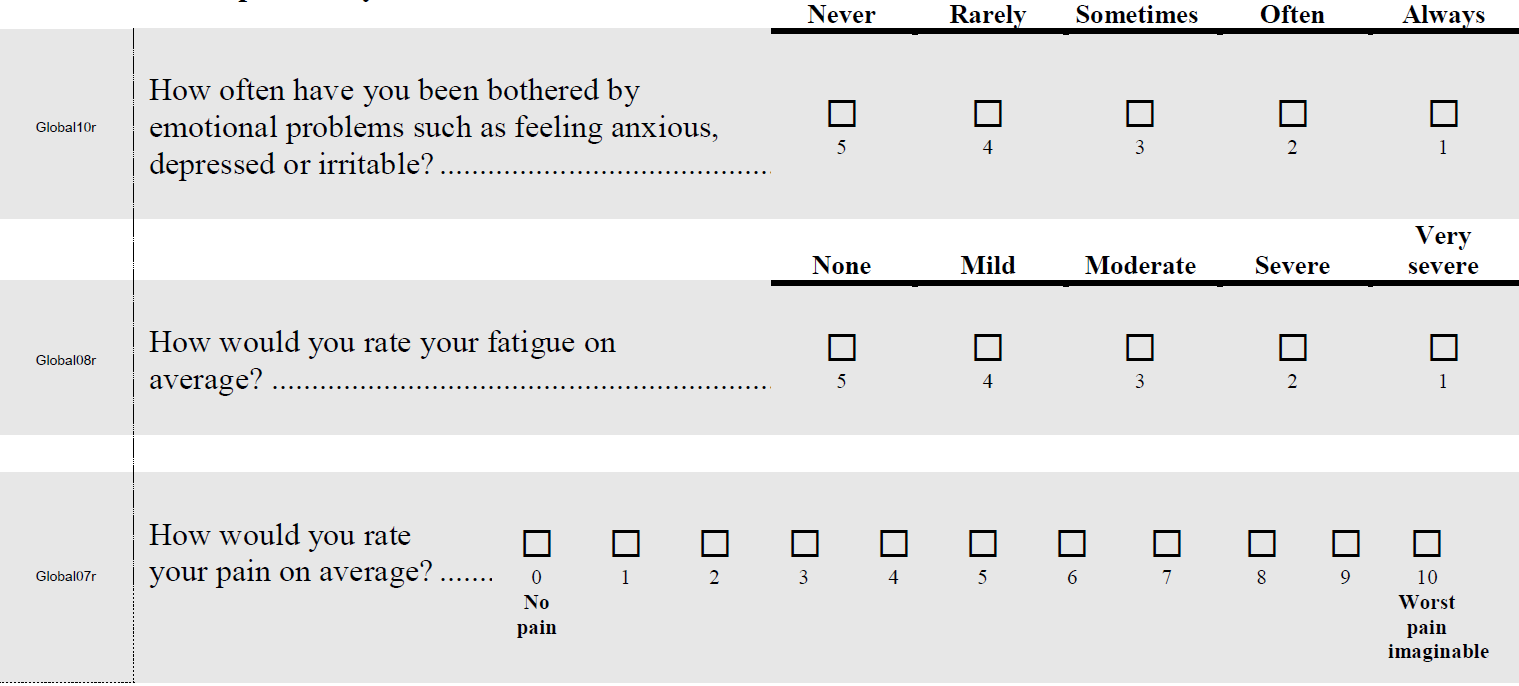


**Patient-Determined Disease Steps (PDDS)**

**Instructions:** Please read the choices listed on this page and choose the one that best describes your own situation. This scale focuses mainly on how well you walk. Not everyone will find a description that reflects their condition. If you feel that your situation is very different from any of the choices below, choose the “U” option and give a brief description of how your condition is different.

**0 Normal:** I may have some mild symptoms, mostly sensory, due to MS but they do not limit my activity. If I do have an attack, I return to normal when the attack has passed.

**1 Mild Disability:** I have some noticeable symptoms from my MS but they are minor and have only a small effect on my lifestyle.

**2 Moderate Disability:** I don’t have any limitations in my walking ability. However, I

do have significant problems due to MS that limit daily activities in other ways.

**3 Gait Disability:** MS does interfere with my activities, especially my walking. I can work a full day, but athletic or physically demanding activities are more difficult than they used to be. I usually don’t need a cane or other assistance to walk, but I might need some assistance during an attack.

**4 Early Cane:** I use a cane or single crutch or some other form of support (such as touching a wall or leaning on someone’s arm) for walking all the time or part of the time, especially when walking outside. I think I can walk 25 feet in 20 seconds without a cane or crutch. I always need some assistance (cane or crutch) if I want to walk as far as 3 blocks.

**5 Late Cane:** To be able to walk 25 feet, I have to have a cane, crutch or someone to hold onto. I can get around the house or other buildings by holding onto furniture or touching the walls for support. I may use a scooter or wheelchair if I want to go greater distances.

**6 Bilateral Support:** To be able to walk as far as 25 feet I must have 2 canes or crutches or a walker. I may use a scooter or wheelchair for longer distances.

**7 Wheelchair/Scooter:** My main form of mobility is a wheelchair. I can stand and

maybe take one or two steps, but I can’t walk 25 feet, even with crutches or a walker.

**8 Bedridden:** Unable to sit in a wheelchair for more than one hour.

**U Unclassifiable:** I don’t fit into any of the categories described above because:

**Neuro-QOL Item Bank v1.0 –Anxiety – Short Form**

**Please respond to each question or statement by marking one box per row.**


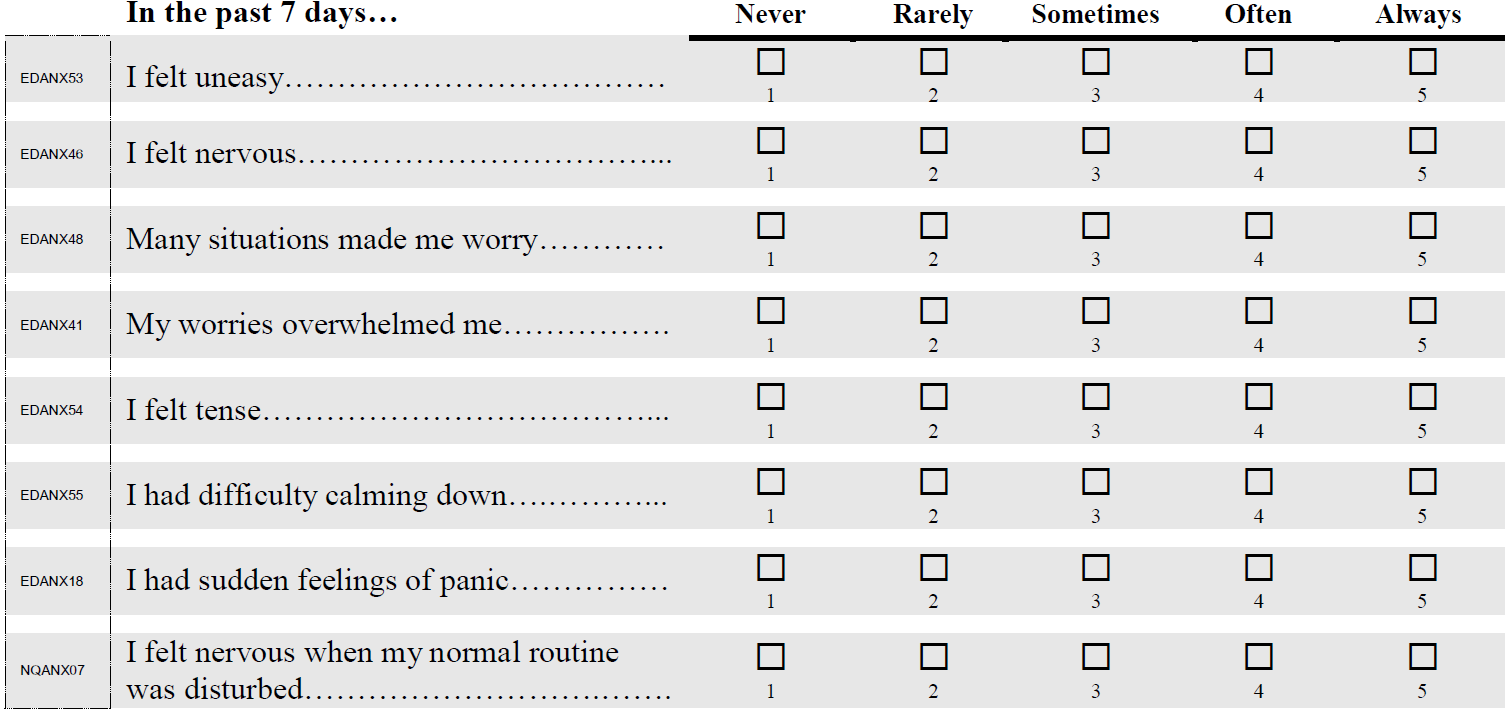


**Additional Demographic Questions**

1. Were you employed on the date of your last infusion? **[If NO, skip to question 4]**

2. **[IF EMPLOYED]**, Did you have to take time off to travel to and receive the infusion?

3. **[IF EMPLOYED]**, How many hours of work did you miss due to traveling to the infusion center and receiving the infusion?

4. Approximately how long did it take to commute to your infusion?

**Post-Infusion PROs (as examples)**

**Additional questions to the pre infusion questions: Home Health Care CAHPS (selected items)**

**1. In the last 2 months of care, how often did home health providers from this agency keep you informed about when they would arrive at your home?**

1 Never

2 Sometimes

3 Usually

4 Always

**2.In the last 2 months of care, how often did home health providers from this agency treat you as gently as possible?**

1 Never

2 Sometimes

3 Usually

4 Always

**3.In the last 2 months of care, how often did home health providers from this agency explain things in a way that was easy to understand?**

1 Never

2 Sometimes

3 Usually

4 Always

**4. In the last 2 months of care, how often did home health providers from this agency listen carefully to you?**

1 Never

2 Sometimes

3 Usually

4 Always

**5. In the last 2 months of care, how often did home health providers from this agency treat you with courtesy and respect?**

1 Never

2 Sometimes

3 Usually

4 Always

**6. We want to know your rating of your care from this agency’s home health providers. Using any number from 0 to 10, where 0 is the worst home health care possible and 10 is the best home health care possible, what number would you use to rate your care from this agency’s home health providers?**

|  | 0 Worst home health care possible | |
| --- | --- | --- |
|  | 1 | |
|  | 2 | |
|  | 3 | |
|  | 4 | |
|  | 5 | |
|  | 6 | |
|  | 7 |  |
|  | 8 |  |
|  | 9 |  |

 10 Best home health care possible

I preferred receiving my infusion at home compared to an outpatient infusion center.

4 Strongly disagree

3 Disagree

2 Neutral

1 Agree

0 Strongly agree

Receiving my infusion at home allowed me to be more productive on the day of the infusion

4 Strongly disagree

3 Disagree

2 Neutral

1 Agree

0 Strongly agree

If COVID-19 risk is minimal/zero (e.g., vaccine available), would you opt to have your next infusion in an outpatient/ambulatory setting or at home?

1 Outpatient/Ambulatory

2 Home

HCAHPS

**1. During your last infusion, how often did your nurse treat you with courtesy and respect?**

1 Never

2 Sometimes

3 Usually

4 Always

**2. During your last infusion, how often did your nurse listen carefully to you?**

1 Never

2 Sometimes

3 Usually

4 Always

**3. During your last infusion, how often did your nurse explain things in a way you could understand?**

1 Never

2 Sometimes

3 Usually

4 Always

**4. During your last infusion, did you get information about what symptoms or health problems to look out for after the infusion?**

1 Yes

2 No

**5. Using any number from 0 to 10, where 0 is the worst experience possible and 10 is the best experience possible, what number would you use to rate this infusion experience?**

 0 Worst experience possible

 1

 2

 3

 4

 5

 6

 7

 8

 9

 10 Best experience possible

**PROMIS 10 – Global Health**

**Please respond to each question or statement by marking one box per row.**


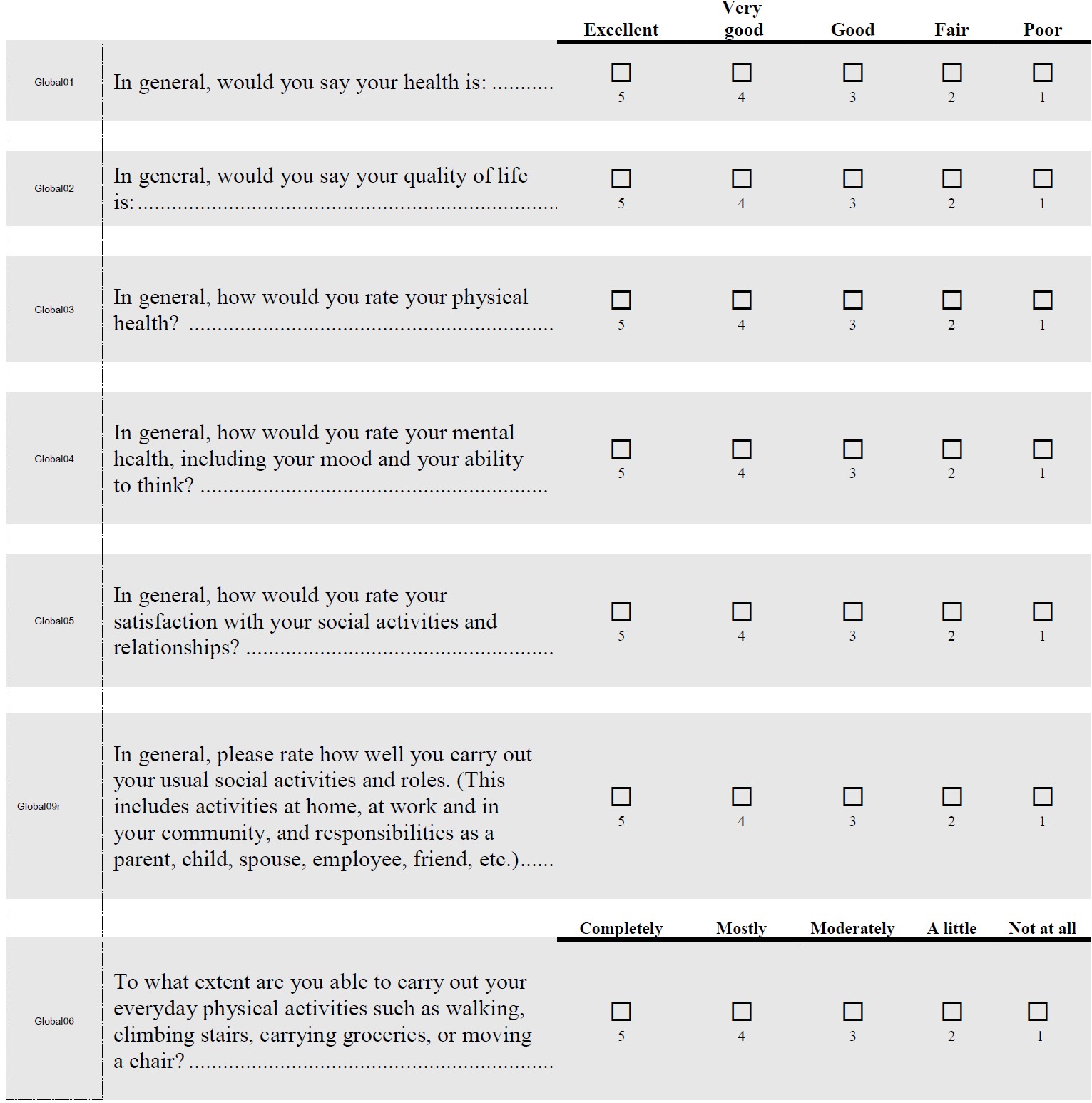


**In the past 7 days…**


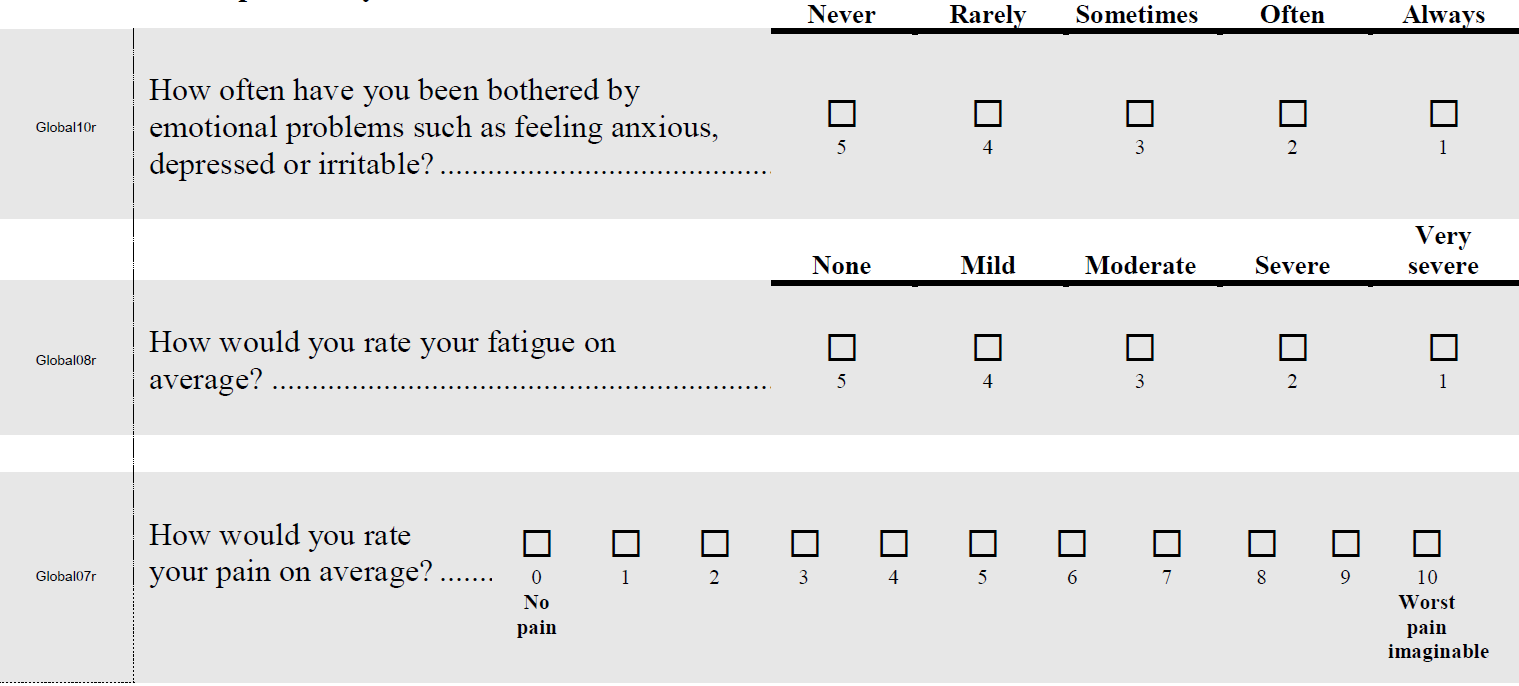


**Neuro-QOL Item Bank v1.0 –Anxiety – Short Form**

**Please respond to each question or statement by marking one box per row.**


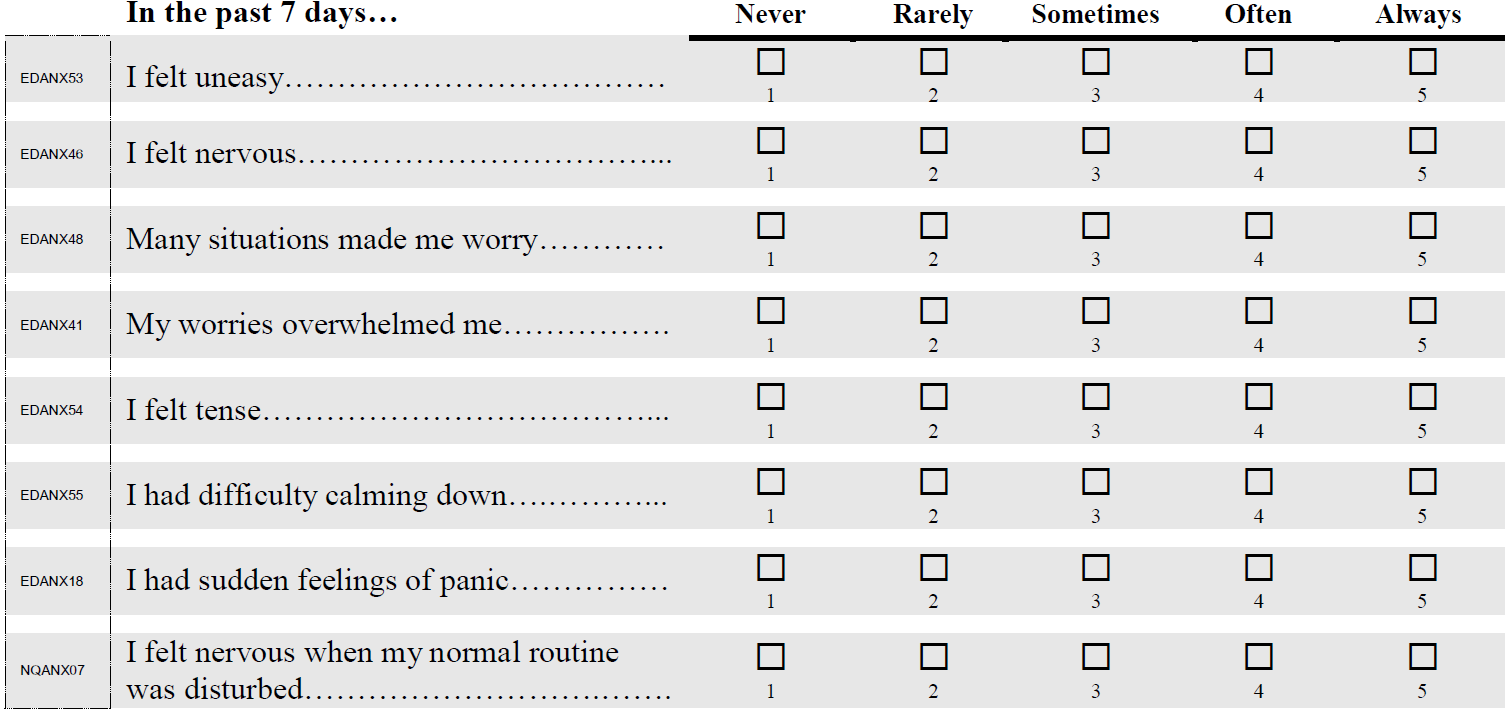


**Additional Questions:**

Overall, receiving my Ocrevus infusion was a good experience.

o strongly disagree

o disagree

o neither agree or disagree

o agree

o strongly agree

I felt confident in infusion nurses administering my infusion.

o strongly disagree

o disagree

o neither agree or disagree

o agree

o strongly agree

I felt respected and safe throughout the infusion.

o strongly disagree

o disagree

o neither agree or disagree

o agree

o strongly agree

I felt comfortable in my surroundings during the infusion.

o strongly disagree

o disagree

o neither agree or disagree

o agree

o strongly agree

I felt worried about possible safety or adverse events during or after the infusion

o strongly disagree

o disagree

o neither agree or disagree

o agree

o strongly agree

If a safety or adverse event were to occur, I felt the medical professionals would be well- equipped to respond appropriately

o strongly disagree

o disagree

o neither agree or disagree

o agree

o strongly agree

The location of the infusion center is convenient to home/work/daily schedule

o strongly disagree

o disagree

o neither agree or disagree

o agree

o strongly agree
